# Echocardiographic assessment of fetal cardiac function in the uterine artery ligation rat model of IUGR

**DOI:** 10.1038/s41390-020-01356-8

**Published:** 2021-01-27

**Authors:** Yichen Dai, Dan Zhao, Ching Kit Chen, Choon Hwai Yap

**Affiliations:** 1 Department of Biomedical Engineering, National University of Singapore, Singapore, Singapore; 2 Department of Ultrasound, Shengjing Hospital of China Medical University, Shenyang, China; 3 Department of Paediatrics, Yong Loo Lin School of Medicine, National University of Singapore, Singapore, Singapore; 4 Department of Paediatrics, Division of Cardiology, Khoo Teck Puat-National University Children’s Medical Institute, National University Health System, Singapore, Singapore; 5 Department of Bioengineering, Imperial College London, London, UK

## Abstract

**Background:**

Intrauterine growth restriction (IUGR) leads to cardiac dysfunction and adverse remodeling of the fetal heart, as well as a higher risk of postnatal cardiovascular diseases. The rat model of IUGR, via uterine artery ligation, is a popular model but its cardiac sequelae is not well investigated. Here, we performed an echocardiographic evaluation of its cardiac function to determine how well it can represent the disease in humans.

**Methods:**

Unilateral uterine artery ligation was performed at embryonic day 17 (E17) and echocardiography was performed at E19 and E20.

**Results:**

Growth-restricted fetuses were significantly smaller and lighter, and had an higher placenta-to-fetus weight ratio. Growth-restricted fetal hearts had reduced wall thickness-to-diameter ratio, indicating left ventricular (LV) dilatation, and they had elevated trans-mitral and trans-tricuspid E/A ratios and reduced left and right ventricular fractional shortening (FS), suggesting systolic and diastolic dysfunction. These were similar to human IUGR fetuses. However, growth-restricted rat fetuses did not demonstrate head-sparing effect, displayed a lower LV myocardial performance index, and ventricular outflow velocities were not significantly reduced, which were dissimilar to human IUGR fetuses.

**Conclusions:**

Despite the differences, our results suggest that this IUGR model has significant cardiac dysfunction, and could be a suitable model for studying IUGR cardiovascular physiology.

**Impact:**

- Animal models of IUGR are useful, but their fetal cardiac function is not well studied, and it is unclear if they can represent human IUGR fetuses.
- We performed an echocardiographic assessment of the heart function of a fetal rat model of IUGR, created via maternal uterine artery ligation.
- Similar to humans, the model displayed LV dilatation, elevated E/A ratios, and reduced FS.
- Different from humans, the model displayed reduced MPI, and no significant outflow velocity reduction.
- Despite differences with humans, this rat model still displayed cardiac dysfunction and is suitable for studying IUGR cardiovascular physiology.

## Introduction

Intrauterine growth restriction (IUGR) is defined as the failure of a fetus to reach its growth potential^1^ and is often caused by placental insufficiency, leading to inadequate nutrients and oxygen supply to the fetus.^2–4^ IUGR occurs in 3–10%^5,6^ of pregnancies, and is one of the major causes of prenatal and perinatal mortality and morbidity, but yet there is currently no proven strategy to prevent or treat it.^7^ There is thus an urgency for improved basic understanding of the disease.

Many studies have shown that IUGR leads to an increased risk of developing cardiovascular diseases in adulthood,^4,8,9^ such as hypertension,^10–12^ hyperlipidemia,^11,12^ and coronary heart disease.^10,13,14^ This is postulated to be a result of fetal programming, where adaptations during fetal stages to growth-restricted conditions disrupted normal development to cause these increased postnatal risks.^12,14–16^ IUGR involves increased vascular resistance in the placenta,^17,18^ and reduced gas exchange with maternal blood leads to hypoxia.^19,20^ These contributors to developmental programming are thought to induce hypertension.^21,22^ The IUGR fetal heart remodels to be more globular in shape,^23^ may be hypertrophied,^24^ and may have altered tissue architecture.^25^ It shows signs of systolic and diastolic dysfunction.^4,26^ There is increased myocardial performance index (MPI),^27^ reduced systolic ejection velocities and ejection force,^28,29^ reduced stroke volume coupled with increased heart rate (HR),^30^ and increased isovolumic relaxation time suggesting poor myocardial relaxation.^24,31^ It is important to improve our understanding of the pathophysiology of these adverse remodeling and dysfunctions, and their impact on postnatal health, to find ways to mitigate the cardiovascular risks.

To this end, animal models of IUGR are very useful, as they can provide samples that are difficult to obtain in human studies, and they allow more interventional investigations. To date, many different IUGR animal models have been established.^32,33^ However, there are very few studies on the fetal cardiac function in these models, in which limited cardiac function parameters were evaluated. There is thus insufficient data on whether the cardiac function of these animal models was similar to those in IUGR human fetuses, and we are unable to assess if they are good models of IUGR cardiac dysfunction to support studies of the consequent cardiac maldevelopment and cardiovascular fetal programming. Thus, in the current study, we performed a detailed investigation of the cardiac function of a rat model of IUGR, which was generated via unilateral uterine artery ligation, with the goal of evaluating how similar they are to IUGR human fetal cardiac function. The rat model may be useful because it can produce multiple fetuses per pregnancy and is easier to handle than large animals.

## Methods

### IUGR rat model

The study was approved by the National University of Singapore Institutional Animal Care and Use Committee, under protocol R18-0962. Six- to twelve-month-old Sprague–Dawley rats (InVivos Pte. Ltd.) were anesthetized with 2–3% isoflurane during the surgery and ultrasound scanning. The surgical creation of this IUGR rat model was adapted from Wigglesworth’s method.^34^ At embryonic day 17 (E17), unilateral ligation of the maternal uterine artery was then performed. The ligation causes obstruction of blood supply to the fetuses on the ligated side, especially those closer to the ligation, and the only source of blood supply to the ligated side was the ovarian artery, resulting in insufficient nutrient and oxygen supply in order to develop growth-restricted fetuses on the ligated side.^34^ Ligation was performed on the side with more fetuses to ensure sufficient a number of surviving growth-restricted fetuses. At E19 and E20, an ultrasound was performed to evaluate the fetal size and cardiac function. After ultrasound scanning at E20, fetuses were harvested for physical measurement of fetal and placental weights, and crown-rump length (CRL).

The fetuses were divided into three groups. Normal fetuses from the nonsurgical dams were used as the control group. Growth-restricted fetuses were from the ligated side of the surgical dams, and were the closest 1–3 fetuses from the ligation site to ensure sufficient growth restriction severity.^35^ Sham fetuses were from the contralateral nonligated side of the surgical dams. There were a total of 16 dams in the surgical group and 10 dams in the control group, and every dam provided 1–3 fetuses for echocardiographic measurements. For weight measurements, more fetuses could be used as this was performed after harvest. In one surgical dam, the ligation did not successfully generate a difference in fetal size between the ligated and nonligated side. This was considered an unsuccessful ligation, and the fetuses were not used for further analysis. Two other animals in which there were postsurgery complications were euthanized and were not included in the analysis. Experiments proceeded until the sample size was at least 5 for every measurement. Dams were randomly selected for control or surgery groups, and the order in which fetuses were ultrasound scanned was randomized.

### Fetal ultrasound

Ultrasound scanning was performed using the Vevo 2100 high-frequency ultrasound machine (FUJI FILM Visual Sonics Inc., Canada). The axial and lateral resolution of the transducers we used were 75 and 165 μm for the MS-250 transducer, and 40 and 80 μm for the MS-550D transducer.

B-mode scans using the MS-250 were used to measure size parameters including biparietal diameter (BPD) and abdominal circumference (AC). The MS-250 was also used for Doppler measurements of the peak umbilical artery velocity (*V*_UA_). The remaining measurements were performed with the MS-550D. After establishing the anatomic axes of the fetus, we located the four-chamber view of the fetal heart. Color Doppler was then used to help locate the atrioventricular valves and the great arteries before taking velocity measurements. Upon location of the required structure, pulsed-wave Doppler was used to measure velocity and time interval parameters. Velocity parameters measured included ascending aorta (*V*_AO_), pulmonary artery (*V*_PA_), mitral valve (*V*_MV_), and tricuspid valve (*V*_TV_). E/A velocity ratios were calculated for both the mitral and tricuspid positions, via E- and A-wave peak velocities. Left ventricle time interval parameters including isovolumetric contraction time (IVCT), isovolumetric relaxation time (IVRT), and ejection time (ET) were used for calculation of left ventricular (LV) MPI (LMPI), which is defined as the sum of IVRT and IVCT divided by ET.^36^ From the four-chamber view, cine B-mode imaging was acquired, and the LV-relative wall thickness (LV-RWT), defined as the ratio of twice the LV free-wall thickness to LV end-diastolic diameter,^37^ was calculated as a measure of LV dilatation. Longitudinal fractional shortening (FS) was calculated for both left and right ventricles, via the manual tracing of the internal boundary of both chambers from the four-chamber view, during end systole and end diastole. FS is defined as the change in internal boundary length normalized by that at end diastole. During all measurements, the observers were blinded to the group assignment.

Intraobserver variability was assessed by repeating measurements for five samples by five times, while interobserver variability was assessed by having two independent observers perform these measurements. The intraclass correlation coefficients were computed, as shown in Table 1, and were found to be satisfactory.

**Table 1:** ICCs of intraobserver and interobserver variability tests.

	Intraobserver ICC	Interobserver ICC
LV-WT	0.960	0.891
LV-EDD	0.986	0.970
LV-RWT	0.953	0.898
LVFS	0.876	0.844
RVFS	0.932	0.872

RWT = 2 × WT/EDD; FS = (EDL − ESL)/EDL.

*ICC* intraclass correlation coefficients, *RWT* relative wall thickness, *FS* fractional shortening, *WT* wall thickness, *EDD* end-diastolic inner diameter, *EDL* end-diastolic length, *ESL* end-systolic length.

### Statistical analysis

The Shapiro–Wilks test was applied to check for data normality. Since all data were found to pass this normality check, we performed the one-tailed *t* test to test for statistical significance. No outliers were excluded from our data.

## Results

### Weight and size

**Fig. 1:**
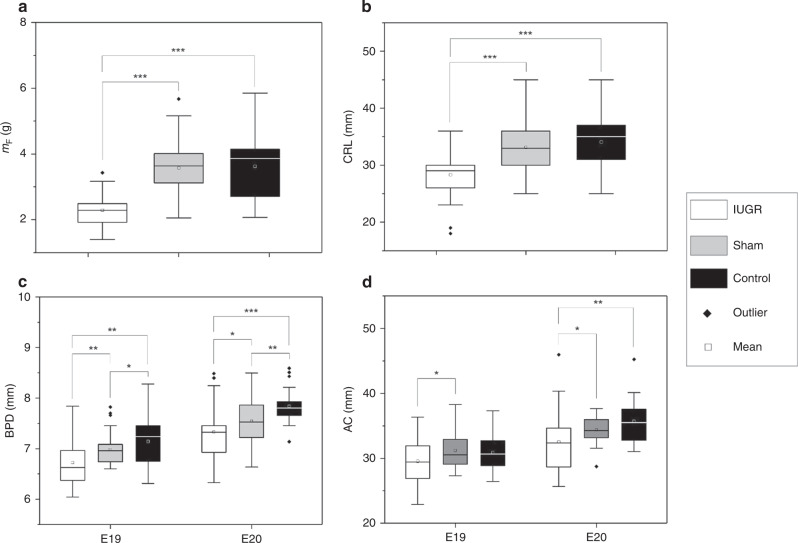
Plots of size parameters of IUGR, sham and normal fetal rats. **A** Fetal weight and **B** crown-rump length (CRL), measured with physical means after harvesting at E20. **C** Biparietal diameter (BPD) and **D** abdominal circumference (AC), measured in vivo with ultrasound at both E19 and E20. *p < 0.05; **p < 0.01; ***p < 0.001. *m*_F_ fetal weight, *m*_p_ placental weight.

Fetal and placental weights measured at the time of harvest at E20 are shown in Fig. 1A, B and Table 2. Growth-restricted fetuses had significantly lower body weight than both sham and control fetuses, and sham fetuses had similar weight with control fetuses. There was a general trend where the growth-restricted fetuses demonstrated a gradient of fetal size according to their distance from the ligation site, similar to previously described.^35^ Furthermore, the average weight of growth-restricted fetuses were lower than the 10th percentile of sham fetal weight distribution. The placental weight of growth-restricted group was significantly lower than that of control and sham groups. The ratio of placental weight to fetal body weight was significantly higher in growth-restricted than sham and control fetuses, while sham and control fetuses shared a similar ratio. Thus, in the growth-restricted group, the effect of growth restriction was more severe on the fetus than on the placenta.

**Table 2:** Fetal and placental weights at E20, measured physically after harvest.

	Control	Sham	IUGR
*m*_F_ (g)	3.62 ± 1.00	3.57 ± 0.89	2.29 ± 0.44^a,b^
*m*_p_ (g)	0.517 ± 0.098	0.547 ± 0.103	0.458 ± 0.066^a,b^
*m*_p_/*m*_F_	0.144 ± 0.035	0.163 ± 0.036	0.214 ± 0.063^a,b^

*m*_F_ fetal weight, *m*_p_ placental weight.

^a^*P* < 0.05, compared to control.

^b^*P* < 0.05, compared to sham.

Fetal size parameters measured using ultrasound are shown in Fig. 1C, D and in Table 3. At E19, growth-restricted fetuses had BPD that were 3.6% smaller than sham, and 5.8% smaller than controls, and by E20, these were 2.9% and 6.5%, respectively. At E19, growth-restricted fetuses had AC that were 5.3% smaller than sham, and 4.3% smaller than controls, and by E20, these were 5.4% and 8.9%, respectively. Growth-restricted fetuses also had significantly shorter CRL compared to control (17.0% shorter) and sham (14.5% shorter) fetuses. While sham fetuses had smaller heads than control fetuses, they had similar abdominal size and CRL.

**Table 3:** Fetal size parameters measured at E19 and E20 in vivo, using ultrasound.

	E19			E20		
	Control	Sham	IUGR	Control	Sham	IUGR
AC (mm)	30.9 ± 2.7	31.2 ± 2.6	29.6 ± 3.6^b^	35.7 ± 3.3	34.4 ± 2.1	32.5 ± 4.8^a,b^
BPD (mm)	7.14 ± 0.48	6.98 ± 0.28^a^	6.73 ± 0.48^a,b^	7.84 ± 0.32	7.54 ± 0.43^a^	7.33 ± 0.50^a,b^
CRL (mm)	^—^	^—^	^—^	34.1 ± 4.6	33.1 ± 4.1	28.3 ± 3.5^a,b^

*AC* abdominal circumference, *BPD* biparietal diameter, *CRL* crown-rump length.

^a^*P* < 0.05, compared to control.

^b^*P* < 0.05, compared to sham.

Overall, these changes demonstrated that unilateral uterine artery ligation significantly affected fetal and placental growth on the ligated side, and successfully generated growth-restricted fetuses. However, the surgery also slightly affected fetal growth on the nonligated side. This could be due to surgical trauma, or abnormal physiology associated with fetal death and resorption on the ligated side, which can affect fetuses on the nonligated side. A further observation could be made that there was an absence of head-sparing effect in this model, since both the abdomen and head reduced in size proportionately for the growth-restricted fetuses.

### Fetal echocardiography

**Fig. 2:**
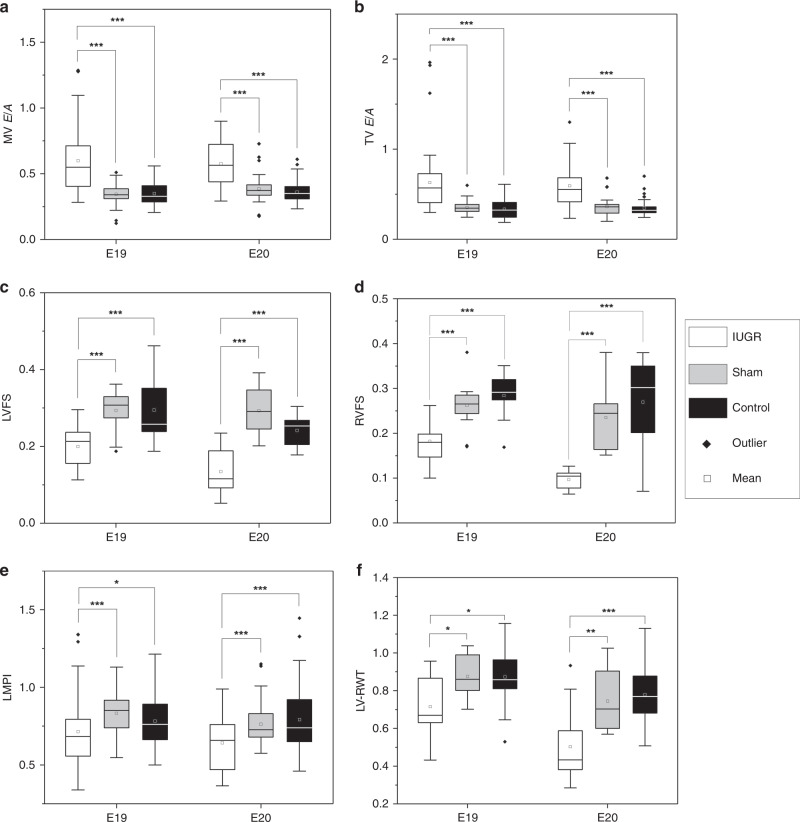
Plots of cardiac function and morphologic parameters that were significantly altered in IUGR fetal rats. **A** Mitral valve (MV) E/A ratio, **B** tricuspid valve (TV) E/A ratio, **C** left ventricular fractional shortening (LVFS), **E**, **D** right ventricular fractional shortening (RVFS) left ventricular myocardial performance index (LMPI), and **F** left ventricular relative wall thickness (LV-RWT) of IUGR, sham and normal fetal rats. *P < 0.05; **p < 0.01; ***p < 0.001.

Fetal echocardiographic measurements are shown in Table 4, and measures that were significantly different in growth-restricted fetal hearts are presented again as plots in Fig. 2. The sample size for all measurements is given in Supplementary Table S1. Doppler velocity measurements in Table 4 showed no consistent trend in ventricular outflow peak velocities among the three groups at E19 and E20. This could imply that reduced outflow velocity was not observed in this animal model, although difficulty in obtaining consistent scanning position and angles of interrogation owing to suboptimal fetal positions could have confounded these differences. Nevertheless, as shown in Fig. 2A, B, growth-restricted fetuses were found to have significantly higher atrioventricular E/A ratios compared to control and sham fetuses, at both mitral and tricuspid positions. This suggested an altered diastolic performance. It is noteworthy that velocity ratio measures are naturally self-normalized, and are thus less susceptible to scanning angle errors.

**Table 4:** Cardiac function and morphologic parameters from echocardiographic measurements.

	E19			E20		
	Control	Sham	IUGR	Control	Sham	IUGR
Peak velocities (mm/s)						
* V*_UA_	143.1 ± 47.4	163.0 ± 42.9	155.8 ± 44.1	203.2 ± 54.7	161.0 ± 64.9^a^	169.6 ± 65.1^a^
* V*_AO_	256.3 ± 68.4	249.1 ± 80.3	284.5 ± 88.9^a,b^	340.4 ± 88.2	321.3 ± 101.2	335.1 ± 97.7
* V*_PA_	299.8 ± 91.4	317.8 ± 110.9	316.1 ± 157.2	412.5 ± 170.4	362.6 ± 111.9	351.7 ± 128.3^a^
Heart rate (b.p.m.)						
	188.4 ± 19.5	187.8 ± 14.9	197.0 ± 75.7	221.6 ± 53.1	224.3 ± 23.0	224.8 ± 70.9
Time intervals (ms)						
IVCT	37.4 ± 13.6	39.6 ± 10.8	38.7 ± 17.6	40.3 ± 11.8	39.2 ± 12.0	30.2 ± 11.4^a,b^
IVRT	63.4 ± 11.2	63.7 ± 14.6	62.3 ± 18.6	53.8 ± 12.3	51.5 ± 8.6	48.3 ± 11.0^a,b^
ET	129.0 ± 11.5	124.7 ± 13.8	138.3 ± 20.8^a,b^	120.7 ± 14.0	119.5 ± 15.5	123.5 ± 12.7
Lengths (mm)						
LV-WT	0.571 ± 0.063	0.487 ± 0.109	0.427 ± 0.067^a^	0.596 ± 0.109	0.527 ± 0.068	0.374 ± 0.096^a,b^
LV-EDD	1.20 ± 0.16	1.11 ± 0.15	1.23 ± 0.20	1.42 ± 0.17	1.37 ± 0.21	1.56 ± 0.22^b^
Ratios						
MV E/A	0.351 ± 0.079	0.345 ± 0.078	0.599 ± 0.236^a,b^	0.362 ± 0.084	0.387 ± 0.104	0.577 ± 0.174^a,b^
TV E/A	0.332 ± 0.109	0.355 ± 0.074	0.629 ± 0.367^a,b^	0.345 ± 0.093	0.364 ± 0.107	0.593 ± 0.253^a,b^
LMPI	0.782 ± 0.160	0.833 ± 0.146	0.715 ± 0.235^a,b^	0.791 ± 0.195	0.762 ± 0.124	0.642 ± 0.164^a,b^
LV-RWT	0.873 ± 0.182	0.875 ± 0.127	0.715 ± 0.171^a,b^	0.779 ± 0.167	0.745 ± 0.168	0.503 ± 0.202^a,b^
RVFS	0.291 ± 0.037	0.258 ± 0.036	0.182 ± 0.052^a,b^	0.267 ± 0.112	0.211 ± 0.053	0.097 ± 0.025^a,b^
LVFS	0.326 ± 0.077	0.290 ± 0.050	0.200 ± 0.052^a,b^	0.251 ± 0.034	0.287 ± 0.063	0.135 ± 0.058^a,b^

LMPI = (IVRT + IVCT)/ET; RWT = 2 × WT/EDD; FS = (EDL − ESL)/EDL.

*UA* umbilical artery, *AO* aorta, *PA* pulmonary artery, *MV* mitral valve, *TV* tricuspid valve, *HR* heart rate, *IVCT* isovolumetric contraction time, *IVRT* isovolumetric relaxation time, *ET* ejection time, *LMPI* left myocardial performance index, *RWT* relative wall thickness, *FS* fractional shortening, *WT* wall thickness, *EDD* end-diastolic inner diameter, *EDL* end-diastolic length, *ESL* end-systolic length.

^a^*P* < 0.05, compared to Control.

^b^*P* < 0.05, compared to Sham.

Ventricular FS was found to be significantly reduced in growth-restricted fetuses compared to control and sham, in both the right and left ventricles (Fig. 2C, D), suggesting systolic dysfunction. In sham fetuses, although there was some reduction in FS compared to control fetuses by E20, the difference did not reach statistical significance.

LMPI (Fig. 2E) was significantly lower in growth-restricted fetuses compared to control and sham fetuses, which is in contrast to most findings in human IUGR studies, as will be discussed below. This was due to an increase in ET at E19, and reduced IVCT and IVRT at E20. Fetal HR was not different between the groups.

Finally, there was also evidence of LV dilatation, as LV-RWT was significantly reduced in growth-restricted fetal hearts compared to both sham and control fetal hearts (Fig. 2F).

## Discussion

The rat model is a popular model to study IUGR physiology.^38–48^ A substantial proportion of these models were generated via uterine artery ligation, while others were generated via diet modifications and hypoxia. These studies were used to understand the effects of fetal growth restriction on various organs, including the kidneys, lungs, and skeletal muscles, but there is significant interest in using them for cardiovascular studies.^38–41,46–50^ A good understanding of the cardiac function in these models will thus be important, as will an assessment of how similar the cardiac function is to that of human fetuses. In our study, we performed such an investigation on the uterine artery ligation rat model, and from our measurements, we found evidence suggesting cardiac dysfunction that was similarly observed in IUGR human fetuses, but we also found essential differences from human fetuses. We discuss our interpretation of the data below, but do not preclude other interpretations.

First, we noted that our rat IUGR model had LV dilatation as evidenced by low LV-RWT values, which is also observed in IUGR human fetuses. Low LV-RWT was used as a measure of ventricular dilatation in human IUGR neonates,^24,51^ and several other studies described dilated ventricles with more globular shape.^11,30^ In our model, however, the dilatation was more severe than that observed in humans. LV-RWT decreased by 25.3% and 40.4% at E19 and E20, respectively, which is higher than the 15.8% decrease in LV-RWT observed in 2-day-old postnatal IUGR humans compared to controls.^52^ In terms of LV wall thickness and diameter, however, while some studies showed hypertrophy,^24,53^ others reported reduced thickness and diameter,^51^ which could be attributed to the varying extent of LV mass reduction during growth restriction. In our study, both sham and growth-restricted LV wall thickness was decreased, but LV diameter was increased in growth-restricted fetuses, compared to control fetuses. Studies in the mouse hypoxia model of IUGR showed LV dilatation and LV mass reduction,^54^ consistent with our results. Ventricular dilatation is thought to be the compensatory response to hypoxia and elevated placental resistance in IUGR,^31^ to enhance cardiac output. However, the prolonged exposure to volume and pressure overload may be the cause of systolic dysfunction.^4^

Significantly reduced LVFS and RVFS of the growth-restricted group in our study suggested the development of systolic dysfunction in growth-restricted rat fetuses. FS is a conventional indicator of systolic performance by assessing the systolic contractility without the need to consider ventricular geometry.^55^ This result was similar to findings in IUGR human fetuses, neonates, and infants, in whom reduced FS or global longitudinal strain were reported as evidence of reduced systolic function.^9,26,56,57^ However, the literature is inconsistent, and some human studies reported no difference in FS between small for gestational age (SGA) and appropriate for gestational age (AGA) groups.^11,24,51,58^ This could be explained by the varying severity of IUGR cases studied. Ozawa et al.^59^ reported nonsignificant decreases of LVFS and RVFS in IUGR human fetuses and explained that this was a result of the inclusion of many mild IUGR cases. Our rat IUGR model can thus be a model for the more severe cohort in this aspect.

In terms of diastolic performance, we interpreted our observations of elevated atrioventricular E/A ratios as a sign of severe diastolic dysfunction. E/A ratios are well-established indicators of diastolic performance, where the E-wave velocity represents passive ventricular filling rate during early diastole, while A-wave velocity represents ventricular filling rate due to atrial contraction during late diastole.^60^ In adults with low severity diastolic dysfunction, impaired ventricular relaxation leads to decreased E-wave velocity to reduce E/A ratio.^61^ However, with increasing severity, elevation in left atrial pressure and preload lead to increased E-wave velocity, leading to a pseudonormalization of E/A ratio.^62^ In severe cases, decreased LV compliance and increased ventricular filling pressure lead to weakened A-wave velocity, causing an elevation of E/A ratio.^63^ Our finding of higher E/A ratio in growth-restricted rat fetuses could thus suggest diastolic dysfunction due to volume overload,^64^ and this is consistent with our observation of ventricular dilatation indicated by a lower RWT. Clinical studies of E/A ratio between SGA and AGA human fetuses, on the other hand, have shown conflicting results, some studies reported an increase in E/A ratio in SGA,^11,23,27,56^ some reported a decrease,^26,65,66^ while others reported no change.^9,24,31,67^ We propose that it can be explained to be the varying extent of diastolic dysfunction, and that our rat model likely represented the severe end of the spectrum.

The LMPI is the central index to assess global cardiac functions that are independent of HR.^68^ In our rat IUGR model, LMPI significantly decreased at both E19 and E20, due to increased ET at E19 and due to decreased IVRT and IVCT at E20. However, studies in humans have reported that LMPI of growth-restricted fetuses, neonates, and infants was either higher than^23,24,27,67^ or similar to^59,69^ AGA controls. High MPI is generally understood to be an indication that the heart is slow to contract and relax, thus indicating that it is failing;^68^ however, during diastolic dysfunction in adult humans, it has been noted that IVRT can shorten when left atrial pressure increases, and that IVRT should be inversely related to LV filling pressures.^62^ The reduced IVRT in our growth-restricted fetal rat hearts may thus be consequent to severe diastolic dysfunction, corroborating with our high E/A ratio measurements, and resulting in low LMPI. Low LMPI in our fetal rat model may also be due to fundamental differences between the physiology between rats and humans, given that we observed higher ET at E19, which is not reported in humans.

In our study, we performed extensive measurements of ventricular outflow velocities, but did not observe specific trends in the results. For example, measurements could be significantly different at one measurement point, but the trend could reverse at the other time point. In human IUGR fetuses, cardiac outflow velocities were reduced,^70,71^ which differed from our rat IUGR model. However, this could be a result of the difficulty in finding consistent scanning position and angle of interrogation in the rat model. Further, in all the rats, normal or diseased, umbilical artery velocities were high during systole and zero during diastole for both E18 and E19 measurements. Thus, unlike in humans, where the absent or reversed end-diastolic velocities can be used to indicate IUGR,^72^ this was not possible in rats. A previous study of another rat IUGR model has found no difference in umbilical arterial flow velocities, corroborating with our findings.^73^

Finally, we noted that all the above cardiac function changed significantly in our growth-restricted rat fetuses, suggesting that they were generally severe, and that the model is suitable for modeling severe disease, but not a mild disease. However, interestingly, the sham group displayed fetal body sizes and cardiac functions slightly reduced from the control group, in between the control and growth-restricted group, likely due to adverse physiology on the ligated side affecting it, and this group may be suitable to be a model for mild growth restriction.

In our study, there are a few limitations. First, the ultrasound scan, due to the manual handling of the transducer can have errors due to imperfect transducer orientation, and noise in the image can lead to variability in locating landmarks. Second, our study did not evaluate other aspects of the animal model, such as molecular markers and gene expressions to confirm fetal growth restriction, but we had used fetal sizes as a surrogate to demonstrate this.

## Conclusion

Overall, we observed that in the unilateral uterine artery ligation IUGR model, growth-restricted fetal rats were similar to IUGR human fetuses in that they suffered from LV dilatation (low LV-RWT), systolic dysfunction (reduced FS), and signs of diastolic dysfunction (high trans-mitral E/A ratios and IVRT). However, growth-restricted rat fetuses also showed decreased LMPI, and did not show significantly decreased outflow velocities, which are different from IUGR human fetuses. Further, growth-restricted rat fetuses did not show signs of the head-sparing effect in their growth. These results suggested that despite the differences, this model can be a relevant model for studying heart dysfunction in the setting of IUGR.

## Supplementary information

Supplementary Text
