# Supplementary material for: Echocardiographic assessment of fetal cardiac function in the uterine artery ligation rat model of IUGR
Source: Pediatr Res. 2021 Jan 27;90(4):801–8. doi: 10.1038/s41390-020-01356-8 (PMC8566221; doi:10.1038/s41390-020-01356-8)
Supplement: Supplementary file 1 — Supplementary Text [file 41390_2020_1356_MOESM1_ESM.docx]

**Supplementary Text**

**Table S1.** Number of fetuses measured for each parameter.

|  | E19 | | | | | E20 | | | | | |
| --- | --- | --- | --- | --- | --- | --- | --- | --- | --- | --- | --- |
| n | Control | Sham | | IUGR | | Control | | Sham | | IUGR | |
| $m_{F}$ | ^—^ | | ^—^ | | ^—^ | | 68 | | 41 | | 41 |
| $m_{P}$ | ^—^ | | ^—^ | | ^—^ | | 68 | | 41 | | 41 |
| $m_{P}/m_{F}$ | ^—^ | | ^—^ | | ^—^ | | 68 | | 41 | | 41 |
| AC | 21 | | 31 | | 34 | | 24 | | 26 | | 28 |
| BPD | 28 | | 25 | | 23 | | 23 | | 20 | | 20 |
| CRL | ^—^ | | ^—^ | | ^—^ | | 68 | | 41 | | 41 |
| $V_{UA}$ | 18 | | 23 | | 24 | | 24 | | 23 | | 26 |
| $V_{AO}$ | 21 | | 22 | | 24 | | 21 | | 25 | | 23 |
| $V_{PA}$ | 14 | | 14 | | 20 | | 18 | | 20 | | 18 |
| IVCT | 28 | | 28 | | 31 | | 32 | | 29 | | 31 |
| IVRT | 28 | | 28 | | 31 | | 32 | | 29 | | 31 |
| ET | 28 | | 28 | | 31 | | 32 | | 29 | | 31 |
| LV-WT | 9 | | 6 | | 9 | | 9 | | 9 | | 12 |
| LV-EDD | 9 | | 6 | | 9 | | 9 | | 9 | | 12 |
| MV E/A | 25 | | 19 | | 26 | | 23 | | 18 | | 25 |
| TV E/A | 16 | | 15 | | 18 | | 15 | | 13 | | 16 |
| LMPI | 28 | | 28 | | 31 | | 32 | | 29 | | 31 |
| LV-RWT | 9 | | 6 | | 9 | | 9 | | 9 | | 12 |
| RVFS | 6 | | 10 | | 13 | | 6 | | 5 | | 5 |
| LVFS | 9 | | 16 | | 16 | | 7 | | 13 | | 11 |
|  | | | | | | | | | | | |
